# Management of cardiopulmonary assist devices in critically ill patients using point-of-care transthoracic echocardiography: a case series

**DOI:** 10.1186/s13089-017-0080-1

**Published:** 2017-11-21

**Authors:** Babar Fiza, Michael Tang, Michael Maile

**Affiliations:** Department of Anesthesiology, University of Michigan Hospitals, 1500 East Medical Center Drive, Ann Arbor, MI 48109-5861 USA

## Abstract

Point-of-care transthoracic echocardiography is increasingly being utilized by the intensive care physicians in the management of hemodynamically unstable patients. However, its use in the management of critically ill patients requiring cardiopulmonary mechanical device support remains to be well described. In this case series, we present two case reports where point-of-care echocardiography was successfully used by the intensive care team in diagnosing and managing problems related to cardiopulmonary assist device malposition.

**Electronic supplementary material:**

The online version of this article (10.1186/s13089-017-0080-1) contains supplementary material, which is available to authorized users.

## Introduction

Traditionally, echocardiography has been performed by cardiologists and cardiac anesthesiologists and, in some hospitals, by sonography technicians with interpretation by cardiologists. Over the past few years, the increasing availability, portability, and better image quality produced by ultrasound machines have resulted in an increased use of transthoracic echocardiogram (TTE) as a point-of-care (POC) ultrasound device. POC TTE is being widely adopted by emergency departments throughout the United States, where its utilization has been demonstrated to achieve great diagnostic capability [1]. In the operative room setting, focused TTE performed by anesthesiologists has been shown to alter management and provide new information in hemodynamically unstable patients [2]. POC echocardiography has even been used as a perioperative screening tool and successfully used in the management of critically ill patients [3, 4]. While the clinical use of POC cardiac ultrasonography continues to expand, this intricate skill requires appropriate education and training, including a comprehensive understanding of its scope and limitations [5].

To date, there are only a few published reports detailing its use in the critical care setting with regard to monitoring of patients with cardiovascular/cardiopulmonary assist devices [6–8]. In the following two case reports, we present the use of POC TTE by the intensive care physicians that resulted in immediate changes in the management of critically ill patients requiring cardiopulmonary mechanical device support.

## Case one: Impella^®^ repositioning

A 52-year-old male with history of chronic obstructive pulmonary disease (COPD), severe mitral regurgitation, severe tricuspid regurgitation, and non-ischemic cardiomyopathy (EF 25%) presented to an outside hospital with altered mental status and acute liver failure in the setting of cardiogenic shock. The patient’s cardiac index was found to be 1.6 L/min at the time of presentation. Despite maximal medical therapy and subsequent placement of an intra-aortic balloon pump (IABP), there was little improvement in the patient’s clinical status. Therefore, an Impella CP^®^ was placed under fluoroscopic guidance in the cardiac catheterization lab. After placement of the Impella device, the patient’s cardiac index improved to 2.4 L/min. An improvement in patient’s mental status and liver function tests were also noted after placement of the Impella.

**Fig. 1:**
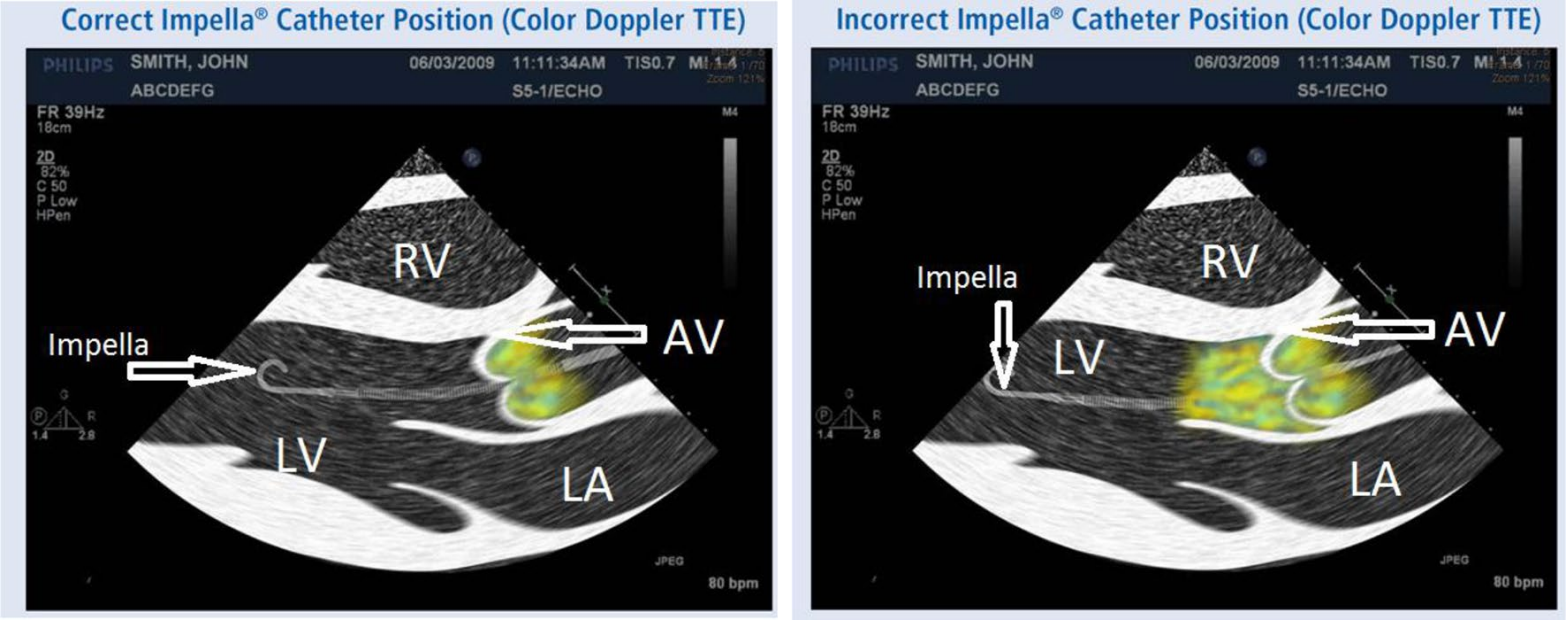
Illustration showing incorrect and correct positioning of Impella^®^ with color flow. Parasternal long-axis view on transthoracic echo (Illustration). In the incorrectly positioned Impella^®^, the outflow is seen below the aortic valve. The catheter is also located too deep in the left ventricle compared to a correctly placed catheter. *AV* aortic valve, *LV* left ventricle, *LA* left atrium, *RV* right ventricle. Image reproduced with permission from Abiomed Corporation

The Impella CP^®^ device is an axial flow type of temporary ventricular assist device that, typically, is placed in the femoral artery and positioned across the aortic valve. This allows it to withdraw blood from the left ventricle and eject it into the aorta. The device is placed percutaneously in patients with cardiogenic shock and provides more hemodynamic support than an IABP [9]. Its initial placement is guided by fluoroscopy or echocardiography to ensure that the inflow area is located approximately 3.5 cm below the aortic valve in the left ventricle and the outflow area is located above the aortic valve (Fig. 1). Typically, blood flows of 2.5–3.5 L/min are achieved through the catheter, which assists low native cardiac output states [10].

**Fig. 2:**
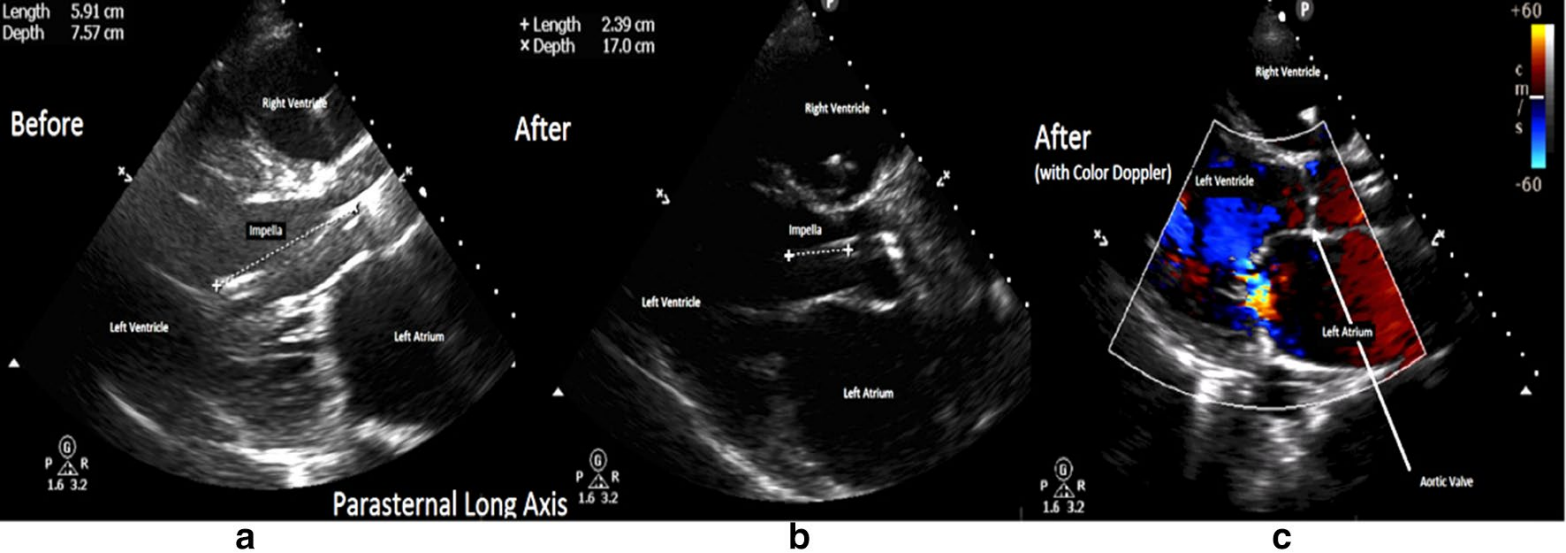
Impella positioning visualized on the parasternal long-axis view on the TTE exam. **a** Images acquired at the time of improper positioning. The Impella^®^ (hyperechoic structure) is located 5.9 cm beyond the aortic valve in the left ventricle. **b** Acquired after repositioning. Notice that the length of the Impella^®^ in the left ventricle is now 2.4 cm beyond the aortic valve into the LV. **c** Acquired after repositioning with color Doppler. Flow at the outflow area can be visualized. The outflow area is now located above the aortic valve

After undergoing placement of the Impella device, the patient was transferred to our cardiovascular intensive care unit (ICU) for evaluation of a left ventricular assist device placement. On arrival, the patient’s hemodynamics were stable and urine was noted to be clear. The placement pressure waveform, a measure of pressure at the outflow of the Impella showed a waveform consistent with an aortic waveform. The motor current, showed good pulsatility, which also indicated correct placement. Next morning, the patient was found to have increasingly dark colored urine, raising concern for hemolysis. Placement waveform signals remained unchanged. A POC TTE performed by the intensive care team showed the Impella^®^ tip to be approximately 5.9 cm below the aortic valve (previously 3.5 cm at the time of placement) in the parasternal long-axis view (Fig. 2). Laboratory workup obtained at this time was consistent with hemolysis and notable for an up-trending lactate dehydrogenase (LDH) and serum-free hemoglobin of 190.3 mg/dL (reference range 1–8 mg/dL for our institution). The cardiac surgery team was notified and subsequently withdrew the Impella by 3 cm. This resulted in resolution of hemolysis as evidenced by clearing of the urine and down-trending LDH and serum-free hemoglobin levels. A repeat POC TTE by the ICU team demonstrated that the catheter was in the correct position, and this was confirmed on color Doppler imaging (Fig. 2; Additional files 1, 2).

## Case two: avalon veno-venous extracorporeal membrane oxygenation cannula malpositioning

A 67-year-old male with history of emphysema, chronic obstructive pulmonary disease, and hypertension presented to our ICU after undergoing a thoracoscopic bilateral lung volume reduction surgery. Postoperatively, the patient developed pneumonia and acute respiratory distress syndrome (ARDS) requiring mechanical ventilation. Despite maximal medical therapy and optimization of mechanical ventilator support, the patient’s oxygenation worsened. On postoperative day seven, the patient’s arterial blood gas analysis was notable for a PaO_2_/FIO_2_ ratio of 40. The patient was placed on pressure control mode of ventilation with inability to achieve greater than 150 mL of tidal volume on inspiratory pressures of 30 mmHg. The decision was made to place the patient on veno-venous extracorporeal membranous oxygenation (VV-ECMO) via the right internal jugular vein with the use of Avalon Elite Bicaval Dual Lumen catheter (MAQUET Holding B.V. and Co. KG, Rastatt, Germany) under direct fluoroscopic guidance. The patient’s oxygenation improved rapidly after VV-ECMO cannulation. ECMO flow was set at 4.0 L/min, sweep at 1 L/min, and ventilation was continued in the same mode as prior to ECMO initiation. Arterial blood gas analysis at this time showed a pH of 7.38, [PaCO_2_] 39, a PaO_2_/FIO_2_ ratio of 170, and a mixed venous oxygen saturation of 65%, indicating improved oxygenation.

VV-ECMO can be used as a salvage therapy for patients with severe or refractory hypoxia [11]. Multiple cannula configurations can be used to take blood from and return it to the venous system. The Avalon Elite Bicaval Dual Lumen Cannula^®^ allows this to be accomplished using a single site. The inflow port of this particular catheter draws blood from both the superior and inferior vena cava. This blood flows into the oxygenator and is then returned through a reinfusion port, which is directed toward the tricuspid valve. Fluoroscopy or transesophageal echocardiography is utilized for proper placement of the catheter to ensure that the distal drainage port is located within the IVC, while the proximal drainage port lies in the superior vena cava [11–13].

**Fig. 3:**
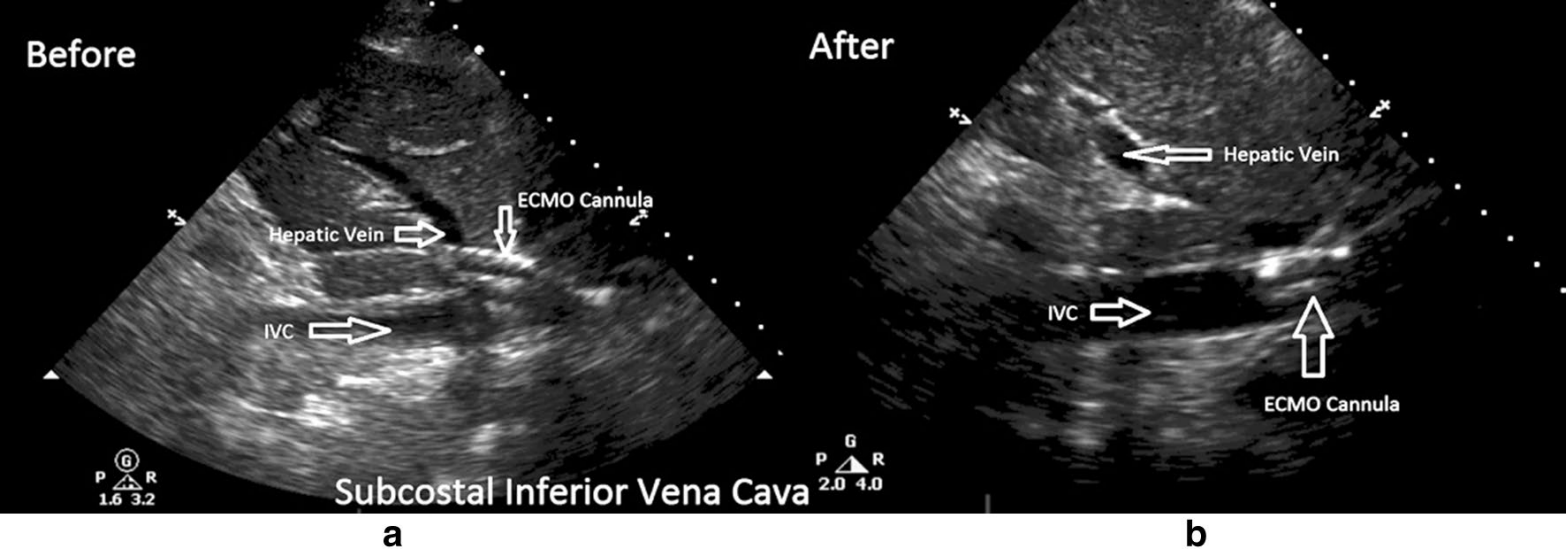
Still Images taken from the subcostal inferior vena cava view on the TTE exam. **a** Image taken during low flows on the ECMO circuit. The ECMO cannula is visualized as a hyperechoic structure located in the hepatic vein. **b** Resolution of low flows on ECMO circuit after catheter repositioning. The ECMO cannula is now located in the lumen of the inferior vena cava. *IVC* inferior vena cava

On ECMO therapy day seven, the patient developed decreased flows, decreased inlet pressures, and chattering of the ECMO tubing. Chattering is vibration of the cannula and tubing that occurs when the drainage cannula abuts the wall of the blood vessel. Chattering most commonly occurs due to inadequate preload due to hypovolemia, or a process causing mechanical obstruction such as tamponade, tension pneumothorax, and abdominal compartment syndrome. Chattering can also occur independent of patient’s volume status due to cannula malposition or excessive ECMO pump speeds. [14] Due to history of aggressive fluid diuresis in this patient, initially fluid boluses were administered to correct the chattering, but the patient continued to have problems with low circuit flows. This resulted in the deterioration in oxygenation with patient developing a pulse oximeter saturation of 80% and a mixed venous oxygen saturation of 43%. A POC TTE was performed by the ICU team at this time to identify the cause of decreased flows. On the POC TTE, the patient had normal right and left ventricular function and there was no pericardial effusion. On the subcostal inferior vena cava (IVC) view, it was noted that the catheter’s distal tip had migrated into the hepatic vein. The Avalon^®^ catheter was immediately withdrawn by 2–3 cm, and repeat POC TTE showed the cannula tip to be located in the inferior vena cava. Correct placement resulted in rapid improvement in ECMO flows and patient oxygenation with pulse oximeter saturation improving to 90% and mixed venous oxygenation saturation to 69% (Fig. 3; Additional files 3, 4, 5).

## Discussion

These two cases illustrate the effective use of POC TTE in rapid diagnosis of clinical problems related to cardiopulmonary mechanical support devices. In the first case, POC echocardiography was used to diagnose Impella malposition. While hemolysis is a documented complication of the Impella device, the underlying mechanism can be related to either the shear stress of the device or improper positioning [15, 16]. The improper positioning of the device can lead to hemolysis as turbulent flow is generated when the outflow of the cannula is too close to the aortic valve or when the outflow of the device is partially below the aortic valve (Fig. 1). Given the different mechanisms that could lead to hemolysis in these patients, it is mandatory to check the cannula position before any therapeutic interventions are undertaken. Unlike the case of malposition, hemolysis related to shear stress would warrant either removal of the device, dependent on the extent of hemolysis, or change in the anticoagulation plan. Bedside TTE thus provides the clinician with the ability to quickly determine if cannula malposition is the cause of hemolysis. The bedside availability of TTE for device placement is recommended by the European Expert Group although very few case reports in literature have described the utilization of bedside TTE for Impella device repositioning [17].

Case one also demonstrates the importance of real-time echocardiographic confirmation of device positioning even when device hemodynamic pressure signals do not display evidence of malposition. The Impella device is fitted with a display screen that continuously monitors device position using a waveform calculated from the pressure difference between the inlet and the outlet areas. Based on the changes in this waveform, a proximal or a distal migration of the device can be detected. As the distal pressures are monitored in an open area above the outlet port, migration of the device outlet area into the aortic valve is possible without a change in waveform [15]. We suspect this to be the case in our patient. In this case, even though the device console indicated appropriate device position, the device malposition was evident on the TTE exam.

The second case highlights the important role of bedside echocardiography in daily management of patients requiring VV-ECMO therapy. Traditionally, VV-ECMO support has been achieved via dual cannulation either through femoro-jugular or femoro-femoral venous cannulation. However, VV-ECMO support can now be achieved via single-vessel cannulation at the internal jugular vein with the use of Avalon Elite Bicaval Dual Lumen Cannula^®^. The advantages of this cannula include less recirculation, improved patient mobility, and decreased risk of infection. However, this catheter like other ECMO catheters when improperly positioned can lead to recirculation, arrhythmias, and a decrease in ECMO flows resulting in hypoxia [7, 8].

As highlighted by our case, echocardiography can assist in determining the cause of inadequate flows and allow clinicians to institute correct therapies. Due to underlying lung pathology, physicians treating these patients generally employ restrictive fluid strategy. This can result in a decrease in ECMO flows due to collapse of the IVC around the cannula. The collapse of the IVC around the catheter combined with signs of hypovolemia can be determined with real-time echocardiography and allow physicians to guide fluid therapy. However, ECMO catheter malpositioning can result in decreased flows as well, in which case the proper therapy would be to reposition the catheter in order to achieve adequate flows. As evidenced by this case, diagnosis of catheter malposition as the cause of decreased flows prevents excessive fluid administration that would otherwise risk worsening of pulmonary function due to volume overload. Additionally, cannula repositioning should always be performed under real-time fluoroscopy or echocardiography guidance as migration of the tip of the cannula into the right ventricle could lead to structural damage and poses risk of arrhythmias [7].

## Conclusion

These two case studies show the effective use of POC TTE by the intensive care physicians to diagnose and manage problems arising from complications related to the use of cardiovascular assist devices. Routine imaging such as plain film X-rays can give us limited information and may not necessarily be sufficient to judge proper positioning. Point-of-care echocardiography proved to be invaluable in the presented cases to diagnose improper positioning, resulting in immediate changes in management.

## Additional files

**Additional file 1.** Parasternal long-axis view obtained at time of improper positioning of the Impella®. The Impella® catheter is located 5.9 cm beyond the aortic valve in the left ventricle.

**Additional file 2.** Parasternal long-axis view obtained after repositioning. The Impella® is now located 2.4 cm beyond the aortic valve into the LV. On color Doppler interrogation flow at the outflow site is now located in the aorta.

**Additional file 3.** On subcostal inferior vena cava view the ECMO cannula is noted to be located in the hepatic vein.

**Additional file 4.** Subcostal inferior vena cava view after repositioning of the ECMO cannula. The cannula is now located within the IVC.

**Additional file 5.** Subcostal inferior vena cava view with color flow Doppler interrogation. Flow is seen in the hepatic vein and the ECMO cannula is located within the IVC.

## Abbreviations

TTE: transthoracic echocardiography
POC: point of care
COPD: chronic obstructive pulmonary disease
IABP: intra-aortic balloon pump
EF: ejection fraction
cm: centimeter
ICU: Intensive Care Unit
LDH: lactate dehydrogenase
mg: milligram
dL: deciliter
AV: aortic valve
LV: left ventricle
LA: left atrium
RV: right ventricle
ARDS: acute respiratory distress syndrome
PaO_2_: partial pressure of oxygen in arterial blood
FiO_2_: fraction of inspired oxygen
mm Hg: millimeters of mercury
PaCO_2_: partial pressure of carbon dioxide in arterial blood
VV-ECMO: veno-venous extracorporeal membranous oxygenation
ECMO: extracorporeal membrane oxygenation
IVC: inferior vena cava
